# Primary hepatic carcinosarcoma: a case report with insights from retrospective analysis of clinical characteristics and prognostic factors

**DOI:** 10.3389/fmed.2024.1470419

**Published:** 2025-01-10

**Authors:** Zonglei Zhao, Tao Wang, Zhexuan Sun, Xuefeng Cao, Xingyuan Zhang

**Affiliations:** Department of Hepatobiliary Surgery, Binzhou Medical University Hospital, Binzhou, China

**Keywords:** primary hepatic carcinosarcoma, liver tumor, risk factors, prognosis, case report, nomogram

## Abstract

Primary hepatic carcinosarcoma (HCS) is an extremely rare malignant tumor with carcinomatous and sarcomatous elements. Few reported cases of HCS exist, especially with sufficient records to describe imaging and pathological features, making the diagnosis, treatment, and prognosis of HCS a significant challenge for physicians. Here, we report a case of HCS with spontaneous rupture as the initial symptom in a 77-year-old elderly male who was admitted with right upper abdominal pain for 8 days. The computed tomography enhancement scan revealed one intrahepatic enhancement with mixed density and a massive, enhanced shadow located mainly outside the liver. We performed a hepatectomy of segment 4 through a laparotomy. The postoperative pathology results demonstrated HCS. The patient recovered smoothly and was discharged after surgery. However, the patient experienced a recurrence and died 5 months after surgery. This case underscores the importance of identifying high-risk populations and personalized treatment strategies in HCS cases.

## Introduction

Primary hepatic carcinosarcoma (HCS) is an extremely rare malignant tumor characterized by a mixture of carcinomatous components (either hepatocellular carcinoma or cholangiocarcinoma) and sarcomatous components (such as spindle cells or differentiation into bone or cartilage) (1–3). The incidence of HCS is very low, with reports indicating that it accounts for less than 1% of all malignant liver tumors; it is more commonly observed in middle-aged and elderly patients, with a slightly higher prevalence in males than in females (4–7). However, due to the rarity of cases and the limited literature, the exact epidemiological features of HCS remain unclear.

The histopathological feature of HCS is that malignant epithelial and mesenchymal components can be observed simultaneously in the tumor under a microscope (7). Immunohistochemistry further aids in the diagnosis: carcinoma components express epithelial markers, whereas sarcomatous components express mesenchymal markers (8–11). However, due to the histological heterogeneity of HCS and the lack of specific imaging features, most reported cases are diagnosed postoperatively based on pathological examination.

This report presents the diagnosis and treatment of a HCS patient. Additionally, we conducted a retrospective analysis of clinical data from published HCS cases in PubMed and CNKI databases, aiming to identify independent risk factors affecting postoperative prognosis. We constructed and validated a nomogram model to effectively guide clinical diagnosis and treatment and help patients with HCS and their physician formulate individualized clinical decisions.

## Case report

A 77-year-old male patient was admitted to the hospital with a chief complaint of right upper abdominal pain that had persisted for 8 days, the etiology of which was unclear. The discomfort was primarily located in the upper right abdomen and was characterized by intermittent sharp pain, with symptoms worsening at night. The patient denied any history of abdominal trauma, although he had been diagnosed with chronic hepatitis B (CHB) at least 30 years prior. However, no antiviral therapy had been administered prior. The patient married a spouse free of physical ailments at an appropriate age, and they had three sons, with their second son dying of primary liver cancer. Upon physical examination, the patient’s blood pressure was within the standard value range, the conjunctiva was not pale, and jaundice was not observed in the sclera. No epigastric tenderness, rebound tenderness, ascites, or lower extremity edema was observed. The level of hepatitis B virus deoxyribonucleic acid (HBV-DNA) was 783 IU/L. Moreover, hepatitis B surface antigen (HBsAg), hepatitis B e antibody, and hepatitis B core antibody were also positive. Other blood tests and tumor marker tests revealed no significant abnormalities.

**Figure 1:**
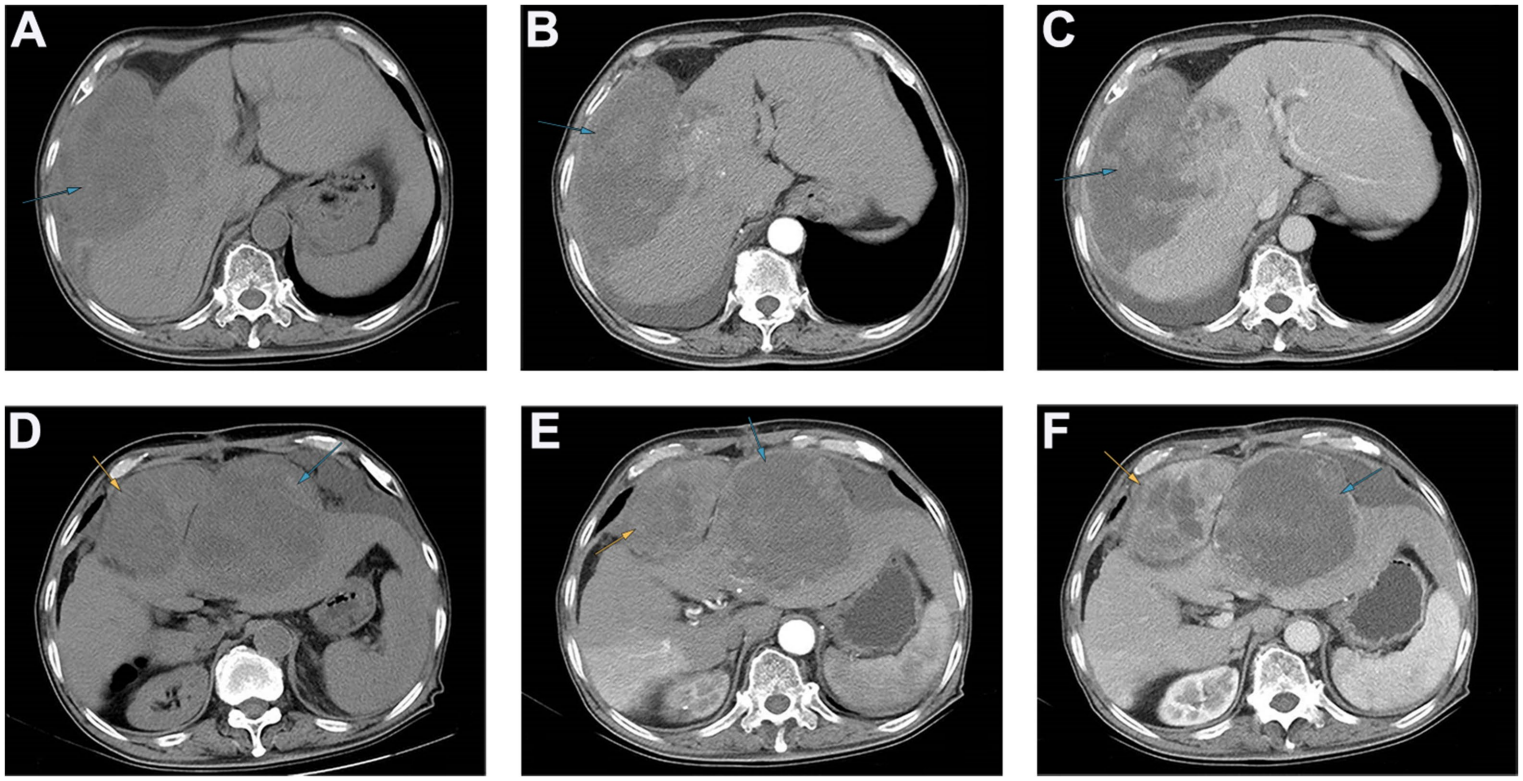
The results of abdominal CT scan of patients with preoperative and postoperative recurrence. **(A–C)** Preoperative abdominal CT revealed that an exogenous mass with a diameter of approximately 16 cm can be seen in the fourth segment of the liver, protruding toward the right diaphragm and invading the diaphragm, and the tumor surface was ruptured. **(D–F)** Postoperative abdominal CT revealed that two areas of low enhancement in the surgical region, with clear boundaries. The larger region was approximately 15 cm, and contained multiple necrotic regions.

Following admission, the patient underwent an imaging examination. A computed tomography (CT) enhancement scan revealed one instance of intrahepatic enhancement with mixed density and a massive, enhanced shadow located mainly outside the liver (Figures 1A–C). In conjunction with the patient’s imaging findings and history of CHB, the patient was initially diagnosed with primary hepatocellular carcinoma with spontaneous ruptured bleeding. Radical resection of the tumors was recommended as a means of controlling the hemorrhage and prolonging survival.

**Figure 2:**
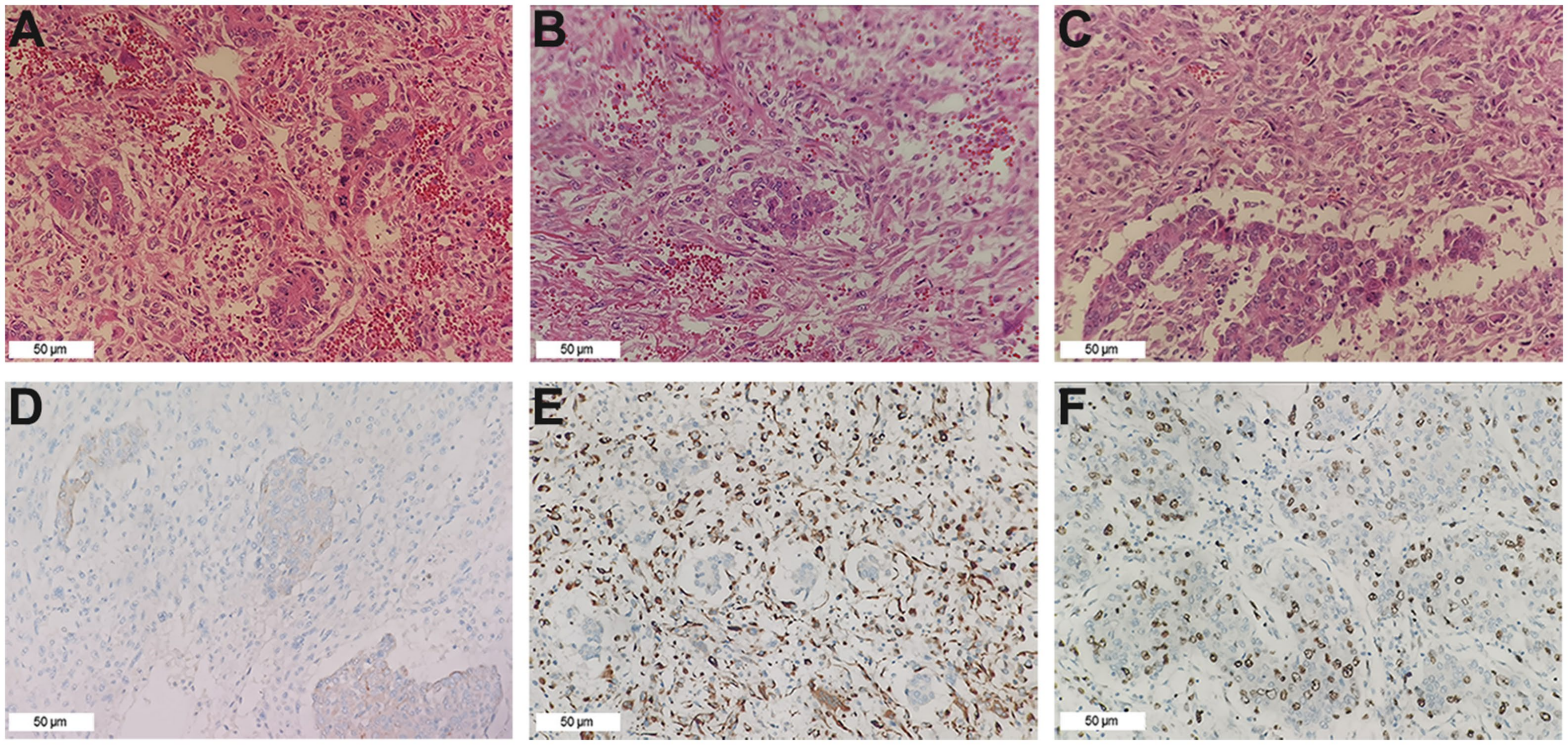
Pathological findings. **(A)** The cholangiocarcinoma component (HE ×200). **(B)** The sarcoma component consists mainly of fusiform cells (HE ×200). **(C)** Border area between cholangiocarcinoma and sarcomatoid components (HE ×200). **(D)** CK7 was positive in the tumor (IHC ×200). **(E)** Vimentin was positive in the tumor (IHC ×200). **(F)** The expression of Ki-67 in the tumor cells was approximately 45% (IHC ×200).

After the above examination, surgical contraindications were ruled out, and a laparotomy was performed. During the operation, a large amount of noncoagulative blood was observed in the abdominal cavity, with a volume of approximately 500 mL, mainly under the left and right diaphragm and pelvic cavity. After draining the accumulated blood, small nodular liver cirrhosis was observed. In addition, an exogenous mass with a diameter of approximately 16 cm can be seen in the fourth segment of the liver, protruding toward the right diaphragm and invading the diaphragm, and the tumor surface was ruptured. Further investigation revealed that the tumor base was relatively small, so it was decided to perform a partial hepatectomy and intraperitoneal chemotherapy. The specimen was sent for pathology testing. Unexpectedly, the postoperative pathology results revealed massive necrotic primary hepatic carcinosarcoma but no evidence of intravascular tumor thrombosis, indicating that the microvascular invasion assessment was M0. Immunohistochemical analyses revealed that the tumor cells were negative for AFP, HepPar-1, CEA, GPC-3, CK19, CK20, Syn, CgA, and CD56 but positive for CK7, Vimentin, and Ki-67, with a Ki-67 expression level of approximately 45% (Figure 2).

The patient recovered well and was discharged smoothly. However, the patient was readmitted to the hospital just 5 months after the surgery due to upper abdominal pain. Enhanced CT of the upper abdomen revealed two areas of low enhancement in the surgical region, with clear boundaries. The larger region was approximately 15 cm, and contained multiple necrotic regions (Figures 1D–F). Based on the patient’s medical history, tumor recurrence was considered, and rapid growth of the tumor rendered surgical intervention unfeasible. Chemotherapy and local interventional palliative therapy were recommended. However, the patient’s family refused further treatment, and the patient was discharged. Just 9 days after discharge, the patient passed away at home. Survival time after the operation was only 5 months.

## Methods

### Clinical data and data extraction

Two independent reviewers (ZZ and XC) conducted literature searches separately, initially screened titles and abstracts according to the requirements, and included qualified articles after reading the full text. In case of disagreement, the third reviewer (TW) will make the final decision. The two investigators independently extracted data from the eligible literature into the Microsoft Excel spreadsheet and extracted the following data according to the predesigned forms: first author name, sex, age, maximum tumor diameter, cirrhosis, capsule, etc. Inclusion criteria: postoperative pathological confirmation of HCS and availability of relatively complete clinical data.

### Risk of bias assessment

Two researchers (ZZ and XC) independently evaluated the risk of bias in the studies, and disagreements were resolved in consultation with the third researcher (TW). The Cochrane Risk of Bias (RoB) 2.0 tool was used to determine the adequacy of the randomization process, deviations from the intended interventions, missing outcome data, measurement of the outcome, selection of the reported result, and the overall RoB for each study.

### Variable selection and nomogram construction

This study collected the following variables: continuous variables included age and maximum tumor diameter; dichotomous variables included sex, cirrhosis, capsule, HbsAg, anti-HCV, AFP, CEA, and CA19-9. The independent risk factors were incorporated into the column chart model to predict the prognosis of HCS. The calibration curve was drawn using the Bootstrap method with 1,000 resampling iterations to validate the accuracy of the model. The concordance index (*C*-index) and area under the receiver operating characteristic curve (ROC-AUC) were used to measure discrimination. Calibration and clinical utility were assessed using calibration and decision curve analysis (DCA).

### Statistical analysis

Statistical analysis was performed using SPSS 26.0 and R (version 4.2.3) software. Continuous data were presented as mean ± standard deviation. Group comparisons were conducted using the independent samples t-test or Mann–Whitney *U* test. Survival rates were calculated using the Kaplan–Meier method, and comparisons of survival rates were performed using the log-rank test. Independent prognostic factors were analyzed using the multivariate Cox proportional hazards model, and hazard ratios (HR) with 95% confidence intervals (CI) were calculated. A *p*-value of <0.05 was considered statistically significant.

## Results

This study retrospectively collected clinical data from published cases of HCS, totaling 93 cases, including our reported case. Among these patients, 69 were male (74.19%), and 24 were female (25.81%), with an average age of 57.73 ± 12.85 years. Statistical analysis indicated that HCS is more common in elderly males. Among the 93 patients, 52 (55.91%) were HBV antigen positive, 5 (5.38%) were HCV antibody positive, and 48 (51.61%) were diagnosed with liver cirrhosis. In most cases, the tumors were relatively large, with a maximum diameter of 27 cm and an average diameter of 9.80 ± 4.88 cm. Despite the potential for large tumors, symptoms of HCS are typically nonspecific, with the most common being abdominal pain (59.14%), abdominal distension (17.20%), and asymptomatic presentation (12.90%). Only a few cases were detected through elevated serum tumor markers, such as AFP (45.05%), CEA (5.68%), and CA19-9 (29.21%). In summary, normal levels of tumor markers do not exclude the diagnosis of HCS. CT scans are the most commonly used imaging method for HCS patients. In our study, 70 patients (88.61%) underwent CT scans, 27 patients (34.18%) underwent ultrasound examinations, and 18 patients (22.78%) underwent magnetic resonance imaging. On plain CT scans, HCS typically appears as an irregular low-density mass, with the rare presence of a pseudocapsule and often indistinct borders. In contrast to hepatocellular carcinoma, which is characterized by “rapid enhancement during the arterial phase and rapid washout to baseline density during the venous phase,” primary hepatic carcinosarcoma has mild to moderate enhancement during the arterial phase and low-density imaging in the venous phase. Additionally, we observed extensive necrotic lesions in 76 (82.61%) of the tumor samples. This phenomenon may be related to the rapid growth of the sarcomatous component of HCS, which results in insufficient blood supply to support the tumor, leading to necrosis. According to our statistical results, only 16 patients (17.58%) with HCS tumors were reported to have a capsule, and these patients generally had a relatively better prognosis.

**Figure 3:**
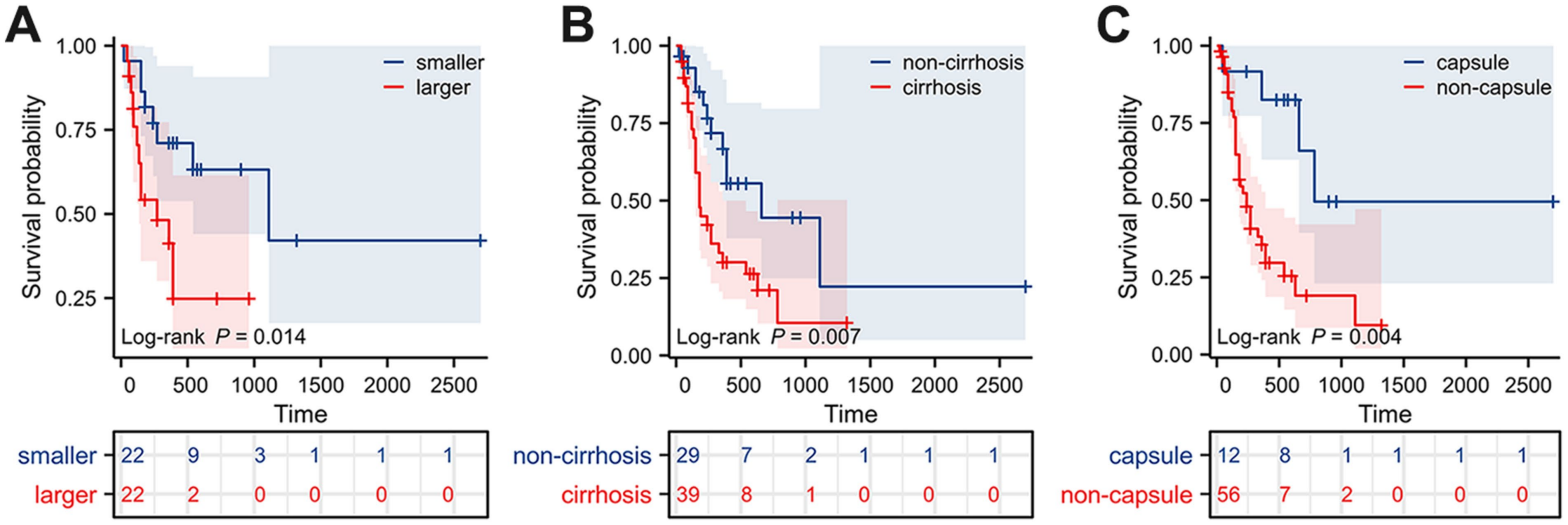
Kaplan–Meier survival curves of HCS patients grouped by different prognostic factors. **(A)** Tumor size. **(B)** Cirrhosis. **(C)** Capsule.

**Figure 4:**
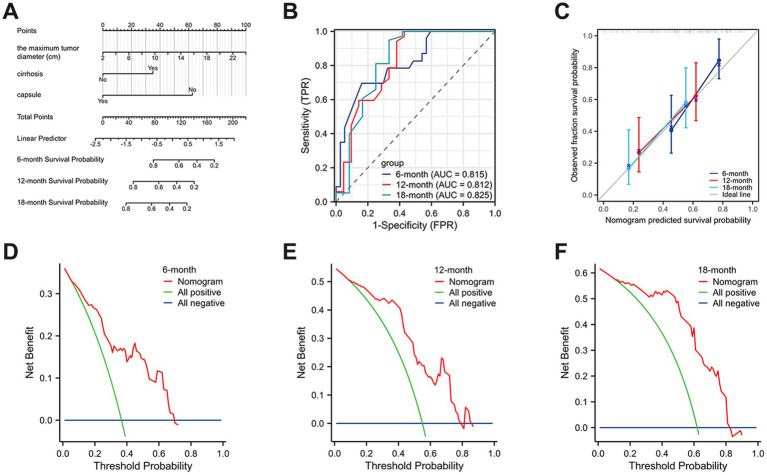
A nomogram prediction model of HCS patients at 6, 12, and 18 months postoperatively. **(A)** The nomogram of HCS patients at 6, 12, and 18 months postoperatively. **(B)** The area under the ROC curve was used to measure the discrimination of the model. **(C)** The calibration curves were plotted to evaluate the precision of the nomogram. **(D–F)** DCA was used to evaluate the clinical utility of the nomogram.

Surgical resection is currently considered the preferred method for treating HCS. In this study, 87 patients (93.55%) underwent surgical resection. However, the prognosis for HCS cases is generally worse than for traditional liver tumors, with a median survival time of only 8 months and an average survival time of 11.52 ± 13.22 months. This poor prognosis may be due to the extensive differentiation and aggressive sarcomatous components of HCS. To explore the risk factors that may affect the postoperative prognosis of HCS patients, we retrospectively collected clinical data from 93 published HCS cases. After excluding cases lacking prognostic information and those not receiving surgical treatment, 68 cases were included in this study. Univariate Cox regression analysis revealed that the maximum tumor diameter, cirrhosis, and lack of a complete capsule affected patient prognosis (*p* < 0.05). Further multivariate Cox regression analysis revealed that maximum tumor diameter (HR = 1.101, 95% CI = 1.026–1.181, *p* = 0.007), cirrhosis (HR = 2.096, 95% CI = 1.054–4.169, *p* = 0.035), and lack of a complete capsule (HR = 3.746, 95% CI = 1.292–10.864, *p* = 0.015) were independent risk factors affecting postoperative prognosis (Table 1). Kaplan–Meier survival analysis was used to evaluate the correlation between these factors and HCS patient prognosis. Based on the tertile method, data were sorted in descending order of tumor size, and the top 33.3% were selected as the large tumor group. In comparison, the bottom 33.3% were selected as the small tumor group to plot the Kaplan–Meier survival curve. The results revealed that prognoses of the large tumor group were significantly worse (*p* = 0.014) (Figure 3A). Patients were categorized into noncirrhotic and cirrhotic groups as well as complete capsule and no-capsule groups. Survival analysis revealed worse prognoses in the cirrhotic and no-capsule groups (*p* < 0.05) (Figures 3B,C). Based on the above analysis, we incorporated independent risk factors into the column chart model to predict the prognoses of HCS. In the nomogram, the tumor’s maximum diameter, cirrhosis status, and capsule integrity were assigned corresponding points, and the total points were obtained by summation. The higher the total points, the worse the prognosis. For example, a maximum tumor diameter of ≤2 cm was assigned 0 points, whereas ≥24 cm was assigned 100 points; cirrhosis was assigned 35 points, and no cirrhosis received 0 points; the absence of an intact capsule was assigned 62.5 points, whereas the presence of an intact capsule received 0 points. For example, a total score of 78 corresponds to a 6-month survival rate of 80% (Figure 4A). To validate the scientific and clinical reliability of the nomogram, a detailed evaluation of discrimination, calibration, and clinical utility was performed. Discrimination was measured using *C*-index and AUC. Calibration and clinical utility were assessed using calibration and DCA. The *C*-index ranges from 0.5 to 1.0, with higher values indicating greater predictive accuracy; the AUC ranges from 0.5 to 1.0, with a value closer to 1 indicating better diagnostic performance of the variable in predicting outcomes. In this study, the nomogram’s *C*-index was 0.734 (95% CI = 0.688–0.780), and the AUC values at 6, 12, and 18 months were 0.815, 0.812, and 0.825, respectively, demonstrating good discrimination (Figure 4B). Subsequently, calibration curves were plotted to evaluate the precision of the nomogram. The white line represents the ideal curve, and the closer the calibration curve is to the ideal line, the higher the predictive accuracy. The calibration curves for the training cohort at 6, 12, and 18 months were close to the ideal line, indicating high predictive accuracy (Figure 4C). Finally, DCA was used to evaluate the clinical utility of the nomogram. The x-axis of the DCA curve represents the threshold probability, whereas the *y*-axis represents the net benefit. When the net benefit curve is above the “all positive” and “all negative” lines at a certain threshold probability, the model has good clinical utility within this threshold probability range. The DCA results in this study showed that for the training cohort at 6, 12, and 18 months, the DCA curves demonstrated positive net benefits within reasonable threshold ranges, suggesting that the probability of patient benefit from the nomogram was significantly greater than those in extreme cases, supporting its high clinical utility (Figures 4D–F).

**Table 1:** Univariate and multivariate Cox regression model analysis of factors affecting overall survival (OS) after radical resection in HCS patients.

Characteristics	Total (*N*)	Univariate analysis		Multivariate analysis	
		Hazard ratio (95% CI)	*p*-value	Hazard ratio (95% CI)	*p*-value
Age	68	1.010 (0.983–1.039)	0.463		
Sex	68				
Female	16	Reference			
Male	52	0.750 (0.366–1.538)	0.432		
The maximum tumor diameter (cm)	68	1.097 (1.025–1.175)	**0.008**	1.101 (1.026–1.181)	**0.007**
Cirrhosis	68				
No	29	Reference		Reference	
Yes	39	2.460 (1.245–4.859)	**0.010**	2.096 (1.054–4.169)	**0.035**
Capsule	68				
Yes	12	Reference		Reference	
No	56	4.274 (1.485–12.307)	**0.007**	3.746 (1.292–10.864)	**0.015**
HBsAg	68				
No	31	Reference			
Yes	37	1.479 (0.769–2.845)	0.241		
Anti-HCV	68				
No	64	Reference			
Yes	4	0.874 (0.210–3.649)	0.854		
AFP (ng/mL)	68				
No	37	Reference			
Yes	31	1.241 (0.665–2.314)	0.498		
CEA (ng/mL)	67				
No	63	Reference			
Yes	4	1.291 (0.386–4.322)	0.679		
CA19-9 (U/mL)	68				
No	49	Reference			
Yes	19	0.845 (0.419–1.706)	0.638		

Bold font indicates that the *p*-value has statistical significance (*p* < 0.05).

### Shortcomings of the model

Although various methods, including the *C*-index, AUC, calibration curve, DCA analysis, and the Bootstrap method, were used to validate the model, the relatively low incidence of HCS has resulted in a limited number of available cases, particularly those with complete clinical data. As a result, this study was unable to divide the data into training and testing sets, which may introduce bias in the model evaluation. Overall, we hope that future reports of additional HCS cases will help refine this model and further deepen our understanding of HCS.

## Discussion

Currently, the definitive diagnosis of HCS still relies on postoperative pathological examination (6, 12). The diagnostic criterion is a malignant epithelial and mesenchymal component in the tumor that can be observed under the microscope (7). Immunohistochemistry can further clarify the diagnosis: in HCS, the carcinomatous component expresses epithelial markers, whereas the sarcomatous component expresses mesenchymal markers (8–10). In our case, HE staining revealed that cholangiocarcinoma cells formed irregular glandular and tubular structures independent of the sarcomatous components, with no clear transition between them. This phenomenon may be related to the origin of HCS (13). The pathogenesis of HCS remains unclear, but the predominant pluripotent stem cell hypothesis suggests that in the liver, pluripotent hepatic stem cells can differentiate into various liver cell types, including hepatocytes, cholangiocytes, and hepatic mesenchymal cells. Under specific conditions, these pluripotent hepatic stem cells undergo mutations and differentiate into both epithelial cells (forming carcinomatous components) and mesenchymal cells (forming sarcomatous components). Consequently, pathological sections reveal distinct carcinomatous and sarcomatous regions with clear demarcation and no evident transition between the two. Liang et al. (6) confirmed the monoclonal origin of carcinosarcomas using two independent clonality assays, supporting the differentiation hypothesis from a single pluripotent stem cell.

Based on the above characteristics, HCS can be distinguished from hepatic sarcomatoid carcinoma, hepatocellular carcinoma, and liver abscess. Hepatic sarcomatoid carcinoma is a primary malignant liver tumor composed of malignant epithelial elements and spindle cell sarcomatoid components (14–16). Its clinical symptoms and histological features are very similar to those of HCS; however, hepatic sarcomatoid carcinoma is a specific type of carcinoma rather than a sarcoma or carcinosarcoma. The sarcomatoid components in hepatic sarcomatoid carcinoma are derived from the transformation of carcinoma cells rather than from true epithelial and mesenchymal tissues (17–19). Thus, clear transition zones can be observed under the microscope. Additionally, immunohistochemical staining revealed that spindle cells in the sarcomatoid region can express epithelial markers. HCC is the most common malignant tumor of the liver (20–24). Its blood supply mainly comes from the hepatic artery, unlike normal liver tissue, which is supplied primarily by the portal vein (25–28). HCC presents rapidly and markedly enhancing dynamic contrast-enhanced imaging, with rapid washout during the portal venous and delayed phases; it may also exhibit a pseudocapsule and is often associated with elevated AFP levels (29–31). In many HCS cases, extensive necrosis is observed, which can easily be confused with liver abscesses. Patients with liver abscesses often have symptoms of fever and chills, and laboratory tests typically reveal significantly elevated white blood cells and neutrophils (32–34). If the abscess has liquefied, imaging may reveal a fluid level within the lesion.

Surgical resection is currently considered the preferred method for treating HCS (6). However, the presence of extrahepatic metastasis, vascular involvement, and surrounding organ invasion can make radical resection extremely difficult. In our case, the patient’s tumor had ruptured before surgery. Despite undergoing surgical resection and 5-FU peritoneal lavage, the patient relapsed 5 months postoperatively. A study reported a 67-year-old Japanese male HCS patient who, after right hepatectomy, received radiotherapy and chemotherapy with doxorubicin and ifosfamide for unresectable lymph node recurrence (35). The results revealed a significant reduction in the size of the lymph node recurrence. The patient achieved 12-month progression-free survival, indicating that this radiotherapy and chemotherapy regimen may prolong the survival of patients with unresectable lesions.

## Conclusion

In conclusion, HCS is a rare malignant tumor with an unclear pathogenesis, for which surgical resection remains the preferred treatment option. However, due to the highly invasive nature of the tumor, the postoperative prognosis for patients is often poor. Overall, our understanding of this disease remains only at the tip of the iceberg, and additional case reports are needed to further characterize this rare tumor.

## Data Availability

The original contributions presented in the study are included in the article/Supplementary material, further inquiries can be directed to the corresponding author.
