# Supplementary material for: Primary hepatic carcinosarcoma: a case report with insights from retrospective analysis of clinical characteristics and prognostic factors
Source: Front Med (Lausanne). 2025 Jan 10;11:1470419. doi: 10.3389/fmed.2024.1470419 (PMC11757265; doi:10.3389/fmed.2024.1470419)
Supplement: Supplementary file 1 [file Table_1.docx]

**Supplementary table.** Clinical data of patients collected from domestic and international HCS case reports

| No | The first author | Sex | Age | Maximum tumor diameter (cm) | Cirrhosis | Capsule | Location | Necrosis | Survival time (months) | Outcome | Symptom | HBsAg | Anti-HCV | AFP(ng/ml) | CEA(ng/ml) | CA19-9(U/ml) | PIVKA(mAU/mL) | Examination | EUS | Treatment | Recurrence |
| --- | --- | --- | --- | --- | --- | --- | --- | --- | --- | --- | --- | --- | --- | --- | --- | --- | --- | --- | --- | --- | --- |
| 1 | Anthony J Freeman | female | 74 | 11 | No | No | Right | No | 12 | alive | 右上腹隐痛 | No | No | Nor(/) | Nor(/) | Nor(/) | / | CT,DSA | Yes(F) | Resection | No |
| 2 | ChunRui Liu | male | 59 | 8 | No | No | Right | Yes | 5 | dead | 无 | No | No | Nor(/) | Nor(/) | Nor(/) | / | US,eCT,PET-CT | No | Resection | Yes |
| 3 | ChunRui Liu | female | 85 | 7.9 | No | No | Right | Yes | 5 | dead | 右上腹痛、发热 | No | No | Rise(25.70) | Nor(/) | Nor(/) | / | US,MRI | Yes(S) | Resection | Yes |
| 4 | ChunRui Liu | female | 76 | 2.5 | Yes | No | Right | No |  |  | 腹痛、腹胀 | Yes | No | Nor(/) | Nor(/) | Nor(/) | / | US | No | Resection | No |
| 5 | Daisuke Kurita | male | 67 | 7.8 | No | No | Right | Yes | 37 | dead | 无 | No | No | Nor(/) | Nor(/) | Nor(/) | Rise(32700.00) | eCT | No | Resection,LND,CHE | No |
| 6 | Fengli Bin | female | 63 | 7.8 | No | No | Left、Right | No | 0.7 | dead | 上腹部钝痛 | No | No | Nor(8.17) | Nor(1.77) | Rise(183.21) | / | CT,MRI | / | Resection | No |
| 7 | Fengli Bin | male | 29 | 8 | Yes | No | Left | No | 21 | dead | 上腹部钝痛 | Yes | No | Nor(5.64) | Nor(0.78) | Nor(2.64) | / | CT | / | Resection | Yes |
| 8 | Fengli Bin | male | 42 | 8 | Yes | No | Left | No | 6 | dead | 上腹部钝痛 | Yes | No | Rise(18.05) | / | Rise(42.27) | / | CT | / | Resection | Yes |
| 9 | Fengli Bin | male | 62 | 9 | Yes | No | Right | No | 11 | dead | 上腹部钝痛 | Yes | No | Nor(2.77) | Nor(1.12) | Nor(9.58) | / | CT | / | Resection | Yes |
| 10 | Hideaki Goto | male | 73 | 3 | Yes | Yes | Left | Yes | 19 | alive | 无 | Yes | No | Rise(120.90) | Rise(8.50) | Rise(173.80） | Rise(240.00) | US,CT,MRI | No | Resection | No |
| 11 | Hirofumi Tazawa | male | 54 | 17 | No | Yes | Left、Right | Yes | 32 | alive | 右上腹痛 | Yes | No | Rise(2980.00) | Nor(2.50) | Nor(2.00) | Rise(1335.00) | eCT | No | Resection,TACE | No |
| 12 | Hong Xie | male | 56 | 23 | Yes | No | Left | Yes | 4 | dead | 腹胀 | Yes | No | Nor(/) | Nor(/) | Nor(/) | / | CT,MRI | No | Resection,TACE | Yes |
| 13 | Inga-Marie Schaefer | male | 76 | 7.5 | No | No | Left | Yes | 8 | alive | / | No | No | Rise(209.00) | Nor(/) | Nor(/) | / | US,MRI | No | Resection | No |
| 14 | Jung Hyeok Kwon | female | 46 | 8 | No | Yes | Left | Yes | 22 | dead | 全身无力 | No | No | Nor(2.10) | Nor(1.50) | Nor(13.20) | / | US,CT,MRI | No | Resection | Yes |
| 15 | Li Jing | male | 45 | 21 | Yes | No | Left | Yes | 2 | alive | 发烧，腹胀 | Yes | No | Rise(/) | Nor(/) | Rise(/) | / | CT | / | Resection | No |
| 16 | Li Jing | female | 69 | 14.5 | No | No | Left | Yes | 13 | dead | 发烧，食欲不振 | No | No | Rise(/) | Nor(/) | Nor(/) | / | CT | / | Resection | Yes |
| 17 | Li Jing | female | 38 | 7.5 | No | No | Right | Yes | 8 | dead | 腹部钝痛 | No | No | Nor(/) | Nor(/) | Nor(/) | / | CT | / | Resection | Yes |
| 18 | Li Jing | male | 61 | 5 | No | No | Left | Yes | 18 | alive | 腹胀 | No | No | Nor(/) | Rise(/) | Rise(/) | / | MRI | / | Resection | No |
| 19 | Li Jing | male | 48 | 7.5 | Yes | No | Right | Yes | 18 | dead | 腹胀 | Yes | No | Nor(/) | Nor(/) | Nor(/) | / | CT | / | Resection | Yes |
| 20 | Li Jing | male | 68 | 10.5 | Yes | No | Right | Yes | 4 | dead | 腹痛、腹胀 | Yes | No | Nor(/) | Nor(/) | Nor(/) | / | CT | / | Resection | Yes |
| 21 | LI LIU | male | 72 | 13 | Yes | No | Left | Yes | 2 | dead | 腹痛 | No | Yes | Rise(7406.00) | Nor(/) | Nor(/) | / | CT,MRI,PET-CT | Yes(F) | Resection | Yes |
| 22 | LiPing Liu | female | 59 | 8 | Yes | No | Right | Yes | 2 | dead | / | No | No | Rise(221.7) | Nor(/) | Nor(/) | / | US,eCT | No | Resection | / |
| 23 | M H Garcez-Silva | male | 40 | 2.6 | Yes | No | Left、Right | No | 5 | dead | 腹水 | No | Yes | Nor(/) | Nor(/) | Nor(/) | / | UN | / | liver transplant | / |
| 24 | Mehmet Çelikbilek | male | 69 | 14 | Yes | No | Left、Right | Yes | 5 | dead | 右上腹痛，体重减轻 | Yes | No | Nor(/) | Nor(/) | Nor(/) | / | CT,US,MRI | Yes(F) | Resection | / |
| 25 | Mingming Zhang | male | 64 | 15 | No | No | Right | Yes |  |  | 上腹痛 | Yes | No | Nor(3.47) | Nor(1.68) | Nor(6.00) | / | CT | / | Resection | / |
| 26 | Mingming Zhang | female | 66 | 7 | No | No | Right | Yes |  |  | 上腹痛 | No | No | Rise(24.87) | Nor(1.51) | Rise(119.86) | / | CT | / | Resection | / |
| 27 | Mingming Zhang | female | 64 | 13 | No | No | Right | Yes |  |  | 上腹痛 | Yes | No | Rise(340.28) | Nor(2.15) | Nor(6.35) | / | CT | / | Resection | / |
| 28 | Seishi JINNOUCHI | male | 55 | 12 | Yes | No | Left | Yes |  |  | 腹胀 | No | No | Nor(/) | Nor(/) | Nor(/) | / | CT,Ga67,DSA | No | No |  |
| 29 | Takatsugu Yamamoto | female | 64 | 22 | No | No | Right | Yes | 3 | dead | 咳嗽 | No | No | Nor(4.60) | Nor(1.60) | Rise(102.00) | Rise(40.00) | CT | No | Resection | Yes |
| 30 | Tsuyoshi YASUTAKE | male | 69 | 20 | No | Yes | Right | Yes |  |  | 腹部钝痛 | No | No | Rise(38.4) | Nor(/) | Nor(/) | Rise(94.00) | CT,MRI | No | Resection | / |
| 31 | XiangMing Lao | female | 50 | 14 | Yes | No | Left | Yes | 2.5 | dead | 腹痛 | Yes | No | Rise(/) | Nor(/) | Rise(/) | / | CT | / | Resection | / |
| 32 | XiangMing Lao | male | 56 | 13 | Yes | No | Right | Yes | 4.5 | dead | 腹痛 | Yes | No | Rise(/) | Nor(/) | Nor(/) | / | CT | / | Resection,RFA | / |
| 33 | XiangMing Lao | male | 68 | 9 | Yes | No | Left | Yes | 6 | dead | 食欲不振 | Yes | No | Rise(/) | Nor(/) | Nor(/) | / | CT | / | Resection | / |
| 34 | XiangMing Lao | male | 55 | 6 | Yes | No | Left | Yes | 6 | dead | 腹痛 | Yes | No | Nor(/) | Nor(/) | Rise(/) | / | CT | / | Resection,RFA | / |
| 35 | XiangMing Lao | male | 40 | 9 | Yes | Yes | Right | Yes | 21 | alive | 体重减轻 | Yes | No | Rise(/) | Nor(/) | Rise(/) | / | CT | / | Radical Resection | Yes |
| 36 | XiaoMin Deng | male | 58 | 4.3 | Yes | No | Right | No | 8 | alive | / | Yes | No | Nor(/) | Nor(/) | Nor(/) | / | CT | No | Resection | No |
| 37 | Xin Zhang | female | 66 | 11 | No | No | Right | Yes | 13 | dead | 腹胀 | No | No | Nor(/) | Nor(/) | Nor(/) | / | / | / | liver transplant,CHE | Yes |
| 38 | Xin Zhang | male | 60 | 6 | Yes | No | Right | Yes | 44 | alive | 无 | Yes | No | Nor(/) | Nor(/) | Nor(/) | / | / | / | Resection,TACE | No |
| 39 | Xin Zhang | male | 66 | 4.6 | Yes | No | Right | Yes | 9 | dead | 右上腹痛 | Yes | No | Rise(/) | Rise(/) | Nor(/) | / | / | / | Resection,RT | Yes |
| 40 | Xin Zhang | male | 46 | 16 | Yes | No | Right | Yes | 24 | alive | 无 | Yes | No | Rise(/) | Nor(/) | Nor(/) | / | / | / | Resection,TACE,CHE | Yes |
| 41 | Xin Zhang | male | 70 | 3 | Yes | No | Right | Yes | 20 | alive | 无 | Yes | No | Rise(/) | Nor(/) | Nor(/) | / | / | / | Resection,TACE | No |
| 42 | Xin Zhang | female | 57 | 9 | Yes | No | Right | Yes | 9 | dead | 右上腹痛、腹胀 | Yes | No | Rise(/) | Nor(/) | Nor(/) | / | / | / | Resection,TACE | Yes |
| 43 | Xin Zhang | male | 39 | 11 | No | No | Right | Yes | 9 | dead | 右上腹痛、发热 | No | No | Nor(/) | Nor(/) | Nor(/) | / | / | / | Resection,TACE | Yes |
| 44 | Xin Zhang | male | 72 | 5.5 | No | No | Right | Yes | 14 | alive | 无 | No | No | Rise(/) | Nor(/) | Rise(/) | / | / | / | Resection,TACE | No |
| 45 | Xin Zhang | male | 73 | 13 | Yes | No | Right | Yes | 3 | alive | 右上腹痛 | Yes | No | Nor(/) | Nor(/) | Nor(/) | / | / | / | liver transplant,CHE | No |
| 46 | Xin Zhang | male | 62 | 6 | Yes | No | Right | Yes | 5 | dead | 腹痛 | Yes | No | Rise(/) | Nor(/) | Nor(/) | / | / | / | Resection | Yes |
| 47 | Xin Zhang | male | 48 | 12 | Yes | No | Right | Yes | 13 | alive | 无 | Yes | No | Rise(/) | Nor(/) | Rise(/) | / | / | / | Resection,TACE,CHE | Yes |
| 48 | Xin Zhang | male | 68 | 13 | No | No | Right | Yes | 6 | alive | 腹胀 | No | No | Rise(/) | Nor(/) | Nor(/) | / | / | / | Resection,TACE,RT | Yes |
| 49 | Xin Zhang | male | 55 | 8 | Yes | No | Right | Yes | 8 | dead | 右上腹痛 | Yes | No | Rise(/) | Nor(/) | Nor(/) | / | / | / | Resectiony,TACE | Yes |
| 50 | YangSheng Lin | male | 72 | 9 | No | Yes | Right | Yes | 16 | alive | 右上腹痛 | No | No | Rise(15164.00) | Nor(/) | Nor(/) | / | US,CT | No | Resection | No |
| 51 | Yi-Jin Gu | female | 58 | 8 | No | No | Left | No |  |  | 发热 | Yes | No | Nor(2.20) | Nor(3.80) | Nor(8.70) | / | eCT | No | Resection | / |
| 52 | Yi-Jin Gu | female | 56 | 4.2 | No | No | Right | No |  |  | 无 | Yes | No | Nor(2.70) | Nor(1.20) | Nor(8.70) | / | eCT | No | Resection | / |
| 53 | Yi-Jin Gu | female | 60 | 3.2 | Yes | No | Right | No |  |  | 腹痛 | Yes | No | Rise(31.40) | Nor(2.80) | Rise(49.10) | / | eCT | No | Resection | / |
| 54 | Yusuke Yamamoto | male | 72 | 4 | No | Yes | Right | Yes | 30 | alive | / | No | Yes | Nor(/) | Nor(/) | Nor(/) | Rise(46.00) | US,eCT | No | Resection | No |
| 55 | Ze Liang | male | 67 | 10 | Yes | No | Left、Right | Yes | 1.5 | alive | 无 | Yes | No | Rise(13.88) | Nor(/) | Rise(41.84) | / | eCT,MRI | No | Resection,RFA,TACE | No |
| 56 | 本案例 | male | 76 | 17 | Yes | No | Left | Yes | 5 | dead | 腹痛 | Yes | No | Nor(1.40) | Nor(1.92) | Nor(4.21) | / | CT | No | Resection | Yes |
| 57 | 陈安 | male | 52 | 14.3 | No | No | Left、Right | No | 9 | alive | 右上腹痛 | No | No | Nor(/) | Nor(/) | Nor(/) | / | US,eCT | No | Resection,CHE | Yes |
| 58 | 陈安 | male | 13 | 6 | No | No | Right | No | 18 | alive | 食欲不振 | No | No | Nor(/) | Nor(/) | Nor(/) | / | US,eCT | No | Resection,CHE | Yes |
| 59 | 陈良 | female | 51 | 10.3 | No | Yes | Right | Yes | 7 | dead | 右上腹隐痛 | No | No | Nor(0.67) | Nor(/) | Rise(38.91) | / | US,eCT | No | Resection | Yes |
| 60 | 丛文铭 | male | 62 | 5.5 | Yes | No | Right | No |  |  | 发热 | No | No | Nor(/) | Nor(/) | Nor(/) | / | US,eCT | No | Resection | / |
| 61 | 邓红琼 | male | 53 | / | / | / | Right | Yes |  |  | 右上腹隐痛 | / | / | Rise(202.00) | Rise(5.40) | / | / | US,eCT | No | Resection | / |
| 62 | 谷江 | male | 7 | 10 | No | No | Left | Yes |  |  | 上腹痛 | No | No | / | / | / | / | / | No | Resection | / |
| 63 | 何度 | male | 60 | 9 | No | No | Left | Yes |  |  | 右上腹痛 | No | No | Nor(2.21) | Nor(2.52) | Rise(1000.00) | / | US,eCT | No | Resection | No |
| 64 | 季峻松 | male | 38 | 9 | Yes | No | Right | Yes | 6 | dead | 发热 | Yes | No | Nor(13.58) | Nor(1.78) | Nor(4.29) | / | eCT,MRI | No | Resection,TACE | Yes |
| 65 | 李日 | male | 56 | 17 | Yes | Yes | Right | Yes | 1.5 | dead | 上腹胀痛 | No | Yes | Nor(8.40) | Nor(/) | Rise(104.90) | / | eCT,MRI | / | Resection,HIPEC | Yes |
| 66 | 连鸿瑞 | female | 69 | 12 | No | No | Left | Yes | 3 | alive | 食欲不振 | No | No | Nor(/) | Nor(/) | Nor(/) | / | US,eCT | No | Resection | No |
| 67 | 梁安民 | male | 35 | 6 | No | Yes | Left | No | 90 | alive | 上腹痛 | No | No | Nor(/) | Nor(/) | Nor(/) | / | RE | No | Resection | No |
| 68 | 卢炜 | male | 68 | 5 | No | 否 | Right | Yes |  |  | 乏力 | No | Yes | Rise(29.46) | Nor(/) | Nor(/) | / | US,eCT | No | Resection | / |
| 69 | 陆力坚 | male | 64 | 9 | No | Yes | Right | Yes |  |  | 右上腹痛 | Yes | No | Rise(/) | Nor(/) | Nor(/) | Rise(/) | eCT | / | Resection | No |
| 70 | 陆力坚 | male | 46 | 12 | Yes | No | Left | Yes |  |  | 右上腹痛 | Yes | No | Rise(/) | Nor(/) | Nor(/) | Rise(/) | eCT | / | Resection | Yes |
| 71 | 陆力坚 | male | 52 | 27 | No | No | Left | Yes |  |  | 上腹痛 | Yes | No | Nor(/) | Nor(/) | Nor(/) | Nor(/) | eCT | / | Resection | Yes |
| 72 | 陆力坚 | male | 61 | 7 | Yes | Yes | Right | Yes |  |  | 右上腹痛 | Yes | No | Nor(/) | Nor(/) | Nor(/) | Rise(/) | eCT | / | Resection | No |
| 73 | 陆力坚 | male | 42 | 15 | Yes | Yes | Right | Yes |  |  | 右上腹痛 | Yes | No | Rise(/) | Nor(/) | Rise(/) | Rise(/) | eCT | / | Resection | No |
| 74 | 陆力坚 | male | 63 | 8.6 | No | No | Right | Yes |  |  | 右上腹痛 | Yes | No | Nor(/) | Nor(/) | Rise(/) | Nor(/) | eCT | / | Resection | Yes |
| 75 | 陆力坚 | female | 52 | 16 | No | No | Left | Yes |  |  | 上腹痛 | Yes | No | Nor(/) | Nor(/) | Rise(/) | Rise(/) | eCT | / | Resection | Yes |
| 76 | 陆孝禹 | male | 56 | 9 | Yes | No | Left | Yes | 3 | dead | 上腹痛 | Yes | No | Rise(300.00) | Nor(/) | Nor(/) | / | US,eCT | No | Resection | Yes |
| 77 | 罗兴喜 | male | 43 | / | No | No | Right | No | 2 | alive | 右上腹痛 | No | No | Rise(139.80) | Nor(/) | Nor(/) | / | US,eCT | No | Resection,CHE | No |
| 78 | 聂长庆 | male | 61 | / | Yes | / | / | / |  |  | 右上腹痛 | Yes | No | / | / | / | / | US,RE | No | No |  |
| 79 | 邱莎莎 | male | 58 | 6 | No | Yes | Right | Yes | 8 | alive | 右上腹痛 | No | No | Nor(1.98) | Nor(/) | Rise(53.71) | / | US,eCT | No | Resection | No |
| 80 | 孙德利 | female | 57 | 7.4 | Yes | No | Right | Yes | 12 | alive | 体重减轻 | Yes | No | Rise(17.00) | Nor(/) | Nor(/) | / | US,eCT | No | Resection | No |
| 81 | 王晓熙 | male | 49 | 17 | Yes | Yes | Left | Yes | 12 | dead | 右上腹痛 | Yes | No | Rise(363.00) | Nor(/) | Nor(/) | / | US,eCT | No | Resection | Yes |
| 82 | 徐海 | male | 57 | 3.8 | No | No | Right | Yes | 13 | alive | 上腹痛 | Yes | No | Nor(/) | Nor(/) | Nor(/) | / | MRI | / | Resection,RFA,TACE | No |
| 83 | 徐海 | female | 67 | 8 | Yes | Yes | Right | Yes | 26.1 | dead | 中上腹痛 | Yes | No | Rise(/) | Nor(/) | Rise(/) | / | eCT | / | Resection,TACE | Yes |
| 84 | 徐海 | male | 60 | 8.4 | No | No | Left | Yes | 12 | dead | 右上腹胀痛 | No | No | Nor(/) | Nor(/) | Rise(/) | / | eCT,MRI | / | Resection | Yes |
| 85 | 徐海 | male | 68 | 7.9 | Yes | No | Right | Yes | 6.3 | dead | 上腹胀痛 | Yes | No | Rise(/) | Nor(/) | Nor(/) | / | eCT | / | Resection,TACE | Yes |
| 86 | 徐海 | male | 55 | 8 | Yes | No | Right | Yes | 3 | dead | 无 | Yes | No | Nor(/) | Nor(/) | Nor(/) | / | eCT | / | Resection,TACE | Yes |
| 87 | 徐海 | male | 52 | 15 | Yes | No | Left | Yes | 3.1 | dead | 腹胀 | Yes | No | Nor(/) | Nor(/) | Nor(/) | / | eCT | / | Resection,CHE | Yes |
| 88 | 杨海鹰 | male | 69 | 5 | No | No | Left | Yes |  |  | 上腹痛 | No | No | Nor(/) | / | / | / | eCT | No | Resection | / |
| 89 | 杨蕗璐 | female | 49 | 4.5 | Yes | No | Left | Yes |  |  | 腹痛、腹胀 | Yes | No | Rise(61.47) | Nor(/) | Nor(/) | / | US,MRI | No | Resection | / |
| 90 | 张恒 | female | 60 | 11 | No | No | Left | Yes | 1 | alive | 右上腹胀痛 | No | No | Nor(2.98) | Rise(6.65) | Rise(2573.00) | Rise(21.00) | US,eCT,MRI | No | Resection | No |
| 91 | 张新龙 | male | 50 | 3.5 | No | No | Left、Right | Yes | 6 | alive | 发热 | No | No | Nor(/) | Nor(/) | Nor(/) | / | US,eCT | No | Resection,TACE | No |
| 92 | 郑学超 | male | 67 | 10.1 | Yes | No | Right | Yes | 1.3 | dead | 体重减轻 | Yes | No | Rise(13.88) | Nor(/) | Rise(41.84) | / | CT,MRI | No | Resection,TACE | Yes |
| 93 | 郑玉荣 | male | 64 | 9 | No | No | Left | Yes |  |  | 上腹痛 | No | No | Nor(/) | / | Rise(75.40) | / | eCT | No | Resection | / |

CT, the computed tomography; eCT, the enhanced computed tomography; MRI, magnetic resonance imaging; US, ultrasound examination; DSA, digital subtraction angiography; PET-CT, positron-emission tomography; Ga67, scanning of radioactive nuclide 67 gallium; RE, radioisotope scanning; EUS, endoscopic ultrasound-guided fine needle aspiration biopsy; LND, lymphadenectomy; CHE, chemotherapy; RFA, radiofrequency Ablation; TACE, transarterial chemoembolization; RT, radiotherapy; HIPEC, abdominal hot perfusion therapy; N, symptomatic treatment.
